# Roles of Birds and Bats in Early Tropical-Forest Restoration

**DOI:** 10.1371/journal.pone.0104656

**Published:** 2014-08-13

**Authors:** Marinés de la Peña-Domene, Cristina Martínez-Garza, Sebastián Palmas-Pérez, Edith Rivas-Alonso, Henry F. Howe

**Affiliations:** 1 Biological Sciences (M/C 066), University of Illinois, Chicago, Illinois, United States of America; 2 Centro de Investigación en Biodiversidad y Conservación, Universidad Autónoma del Estado de Morelos, Cuernavaca, Morelos, Mexico; 3 School of Forest Resources and Conservation, University of Florida, Gainesville, Florida, United States of America; University of Western Ontario, Canada

## Abstract

Restoration of tropical forest depended in large part on seed dispersal by fruit-eating animals that transported seeds into planted forest patches. We tested effectiveness of dispersal agents as revealed by established recruits of tree and shrub species that bore seeds dispersed by birds, bats, or both. We documented restoration of dispersal processes over the first 76 months of experimental restoration in southern Mexico. Mixed-model repeated-measures randomized-block ANOVAs of seedlings recruited into experimental controls and mixed-species plantings from late-secondary and mature forest indicated that bats and birds played different roles in the first years of a restoration process. Bats dispersed pioneer tree and shrub species to slowly regenerating grassy areas, while birds mediated recruitment of later-successional species into planted stands of trees and to a lesser extent into controls. Of species of pioneer trees and shrubs established in plots, seven were primarily dispersed by birds, three by bats and four by both birds and bats. Of later-successional species recruited past the seedling stage, 13 were of species primarily dispersed by birds, and six were of species dispersed by both birds and bats. No later-successional species primarily dispersed by bats established in control or planted plots. Establishment of recruited seedlings was ten-fold higher under cover of planted trees than in grassy controls. Even pre-reproductive trees drew fruit-eating birds and the seeds that they carried from nearby forest, and provided conditions for establishment of shade-tolerant tree species. Overall, after 76 months of cattle exclusion, 94% of the recruited shrubs and trees in experimental plots were of species that we did not plant.

## Introduction

The future of tropical forests will be determined by interplay of climate change, conservation, deforestation, natural succession, and ecological restoration [1]–[3]. Seed dispersal by birds and mammals plays a key role in tropical forest dynamics, and will play a critical role in determining which tree species migrate or vanish in response to changes in land use and climate [3]. Fruit-eating animals that regurgitate, defecate or bury seeds in viable condition are responsible for effective reproduction of most tropical trees [4]–[6]. One unaddressed challenge is to determine the roles that different dispersal agents play in restoration of dispersal processes, which is the issue at hand here [7]. A second challenge is to harness those dispersal agents that most accelerate the process [8], [9]. We address the first challenge and offer a solution to the second for the first 76 months of succession in an agricultural mosaic of pasture, forest remnants, living fences and scattered shade trees with a substantial residual fauna of fruit-eating birds and bats.

Here we address ecological results and consequences of dispersal processes that are relevant to maintaining heterogeneity and accelerating ecological succession in highly altered agricultural landscapes. Our approach uses the interdependence of most rainforest trees on birds and mammals that disperse seeds as a critical phase of tropical tree life cycles (4). The point is to shape foraging routes of fruit-eating animals by providing plantings that offer cover and/or food, and consequently facilitate seed dispersal into fenced habitat islands that serve as stepping stones among forest remnants for “countryside” plants and animals capable of existing in agricultural landscapes [10]. We use well-established recruits of tree species dispersed into experimental exclosures to determine whether fruit-eating birds and bats have comparable roles in promoting forest succession over the first years of ecological restoration.

Our study considers processes of effective dispersal, which involves removal of seeds from parent trees to sites where germination, establishment and survival are possible [11]. Our test of dispersal agency is done in fenced plots that are either left unplanted to simulate natural succession, planted with 12 species of native wind-dispersed trees, or planted with 12 species of native animal-dispersed trees as assisted succession. Patterns of seed fall offer insight into potential composition of regenerating forests [12], but the vast majority of seeds fail to establish as recruits [13]. Relevant to this study, succession in abandoned tropical pastures is slow even if seeds of forest trees arrive [14], [15]. Established recruits are useful predictors of colonization dynamics in early-successional habitats where scattered seedlings are not clumped in dense cohorts near fruiting adults of the same species. In restoration plots far from seed sources, high density-dependent mortality from insects, pathogens, vertebrates and cohort competition is less likely to take a toll on seedlings than in clusters of seeds or seedlings near adults of the same species in forest. Sparsely-distributed seeds and young plants are more likely to succumb to haphazard seed predation by ants and rodents, xeric field conditions, and competition from aggressive grasses.

We test the null hypothesis that fruit-eating birds and bats play comparable roles in promoting tree and shrub recruitment over the first 76 months of experimental restoration in an agricultural-rainforest mosaic in southern Veracruz, Mexico. Some fruit-eating birds and bats forage locally within pastures; others commute long distances to and from feeding areas [16]. Differences in dispersal roles might be expected because fruit-eating bats in agricultural Neotropical landscapes are thought to depend heavily on small-seeded trees, many of which are pioneer trees and shrubs of early succession [17]. In contrast, toucans and other medium-sized to large birds feed on many fruits of trees of late-secondary and primary forest, and carry seeds hundreds of meters to and from feeding sites in forest remnants, isolated trees, and isolated stands of trees [16], [18]. Of particular interest is accumulation of established recruits of pioneer and later-successional tree species that we did not plant in replicated plots that simulate assisted as compared with natural succession.

## Materials and Methods

The study site was in an agricultural mosaic in the Los Tuxtlas Region of southeastern Veracruz, ∼1 km NE of the nearest edge of the Los Tuxtlas Biological Station [19]. Topography is complex, with thin, eroded sandy loam of heterogeneous depth (median 18.5 cm, range 5 ->70 cm) over rocky volcanic deposits [20], [21]. Nearby rainforests, including at least 372 plant species of dbh ≥2.5 cm, are described elsewhere [22], [23]. The landscape is highly fragmented; excluding epiphytes, 81% of plant species occur in fragments of ≤5 ha, and ∼70% occur in ≤5 such fragments [23]. Mean annual temperature and rainfall are 27°C and ∼4900 mm, respectively. Normal dry seasons from March to May sometimes extend as droughts through June (e.g. [24]). The landscape hosts ∼72 species of resident fruit-eating birds and 24 species of fruit-eating bats, most of which frequent forest, forest fragments, and mosaic habitats [7], [25]. No permit is required for plant studies by Mexican nationals on privately-owned land, leased in Veracruz in 2006 from Benito Palacios for 30 years.

The12 ha site where we conducted the study was cattle pasture embedded in an agricultural mosaic of rainforest, isolated trees, and living fences. In August 2006 we established 24 fenced 30×30 m plots separated by 35 m of active cattle pasture arranged in a 3×8 grid (central GPS point 18° 35′ 43.64″ N, 95° 06′ 06.29″ W). Eight exclosures were planted with 12 seedlings of 12 native animal-dispersed species (>10 cm high), and eight with 12 native wind-dispersed species with 12 seedlings each [15], [26]. Eight unplanted controls represented minimal manipulation (fencing) to simulate natural succession on abandoned land. Matrices between exclosures remained closely cropped grasses. Here we compared fenced unplanted controls with fenced plantings of native trees.

Criteria for designations of tree successional status and dispersal category are consistent with general practice [4], [27]. Rationales for departures are outlined in (Appendix S1), along with botanical authorities and plant families (Table S1). Species recruited since 2006 as seedlings, saplings or rapidly-growing trees are listed with references of published reports indicating primary dispersal agents and pioneer or later-successional status (Table S1). An outlier plot of wind-dispersed trees is not included here (deeper, wetter soil with tree growth far ahead of 23 other plots). Recruits ≥10 cm high were recorded every 4–6 months from month 16 (October 2007) through month 76 (January 2013) after cattle exclusion.

This experiment is expected to span 30 years. Prior publications address legacy effects through month 24 after cattle exclusion [28], and recruitment rates as functions of successional status (early or late) and general dispersal mode (animal or wind) through month 60 [15]. Both studies pre-date detectable differences in effects of bats and birds. Other reports address patterns of seed fall [12], soil characteristics [21], mortality of planted seedlings [24] and growth of planted trees over the first 30–42 months [26].

Primary analytical tools are mixed-model repeated-measures randomized-block ANOVAs. Pioneer and later-successional species and recruitment densities are evaluated independently. Independent variables include time (month of census or initial and final census), dispersal agent (birds, bats or both) and treatment (planted or unplanted), with interactions. F-statistics are considered significant when the Bonferroni adjustment is significant at *P*≤0.05. Statistics are accomplished with SAS and Systat 13.

## Results

### Recruitment over time

**Figure 1:**
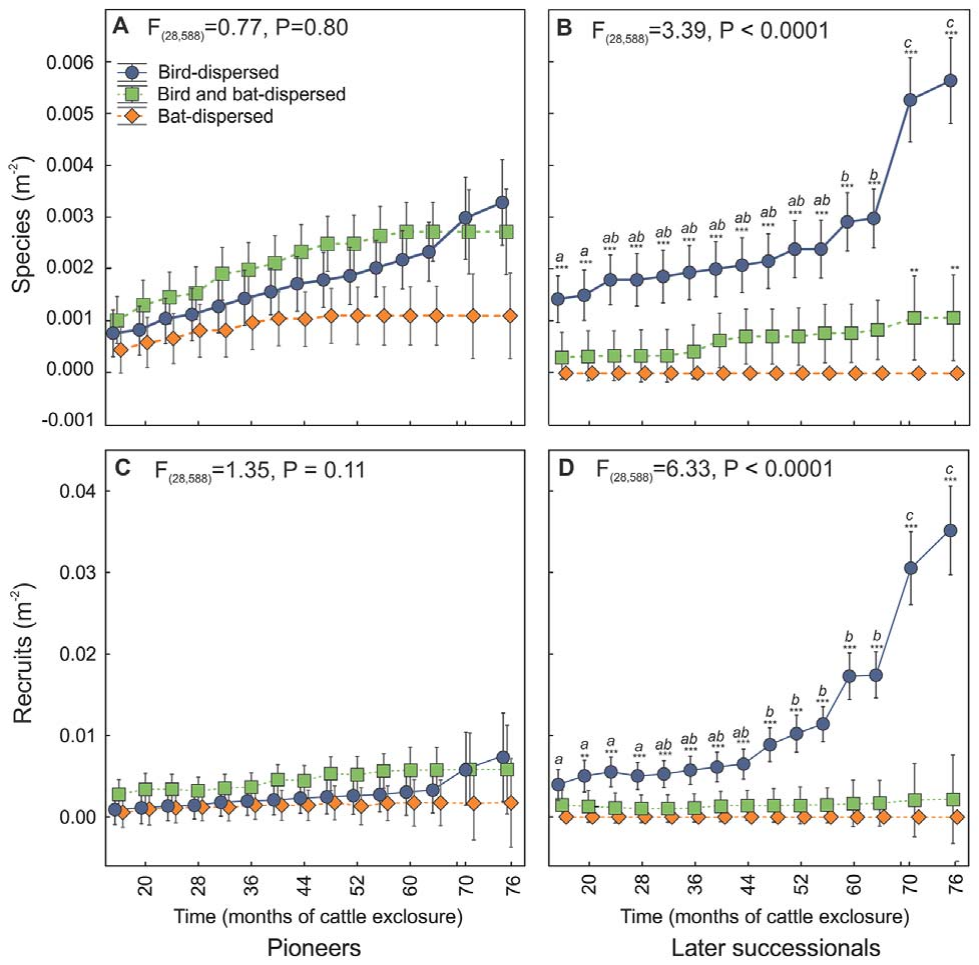
Changes in density of recruited species and individuals by dispersal group over time. Shown are (*A*) pioneer species, (*B*) later-successional species, (*C*) pioneer individuals, (*D*) later-successional individuals. Blue circles indicate primarily bird-dispersed, green squares both bird- and bat-dispersed, and rust diamonds primarily bat-dispersed species. Asterisks indicate differences between dispersal categories in the same time period. ANOVA statistics indicate disperser category by time interactions. Letters show differences for bird-dispersed later-successional trees from one time period to the next using conservative Bonferroni post-hoc tests (*P*≤0.05). Shown are means and 95% confidence intervals.

Distinctively different patterns of recruitment by dispersal category and life history emerged over time (Fig. 1, Tables 1 and S1). Densities of pioneer species increased from 0.0007 species m^−2^ to 0.0023 m^−2^ from 16 to 76 months after cattle exclusion (*P*<0.0001). Significant differences existed in overall contribution of dispersal categories to pioneer-species densities (*P*<0.0001), but interaction of dispersal category of pioneer species densities with time was not significant (Fig. 1*A, P* = 0.80). Individual densities of pioneer recruits increased from a mean of 0.0014 m^−2^ in 2007 to 0.005 m^−2^ in 2013 (*P*<0.0001). Significant overall differences by dispersal category of individual pioneer recruits existed (*P*<0.0001), but again the interaction of dispersal category with time was not significant (Fig. 1*C, P* = 0.11).

**Table 1:** Mixed-model ANOVA of species and recruit densities over time.

	Pioneer			Later successional		
Species density	num.d.f.	den.d.f.	F-value	*P*	F-value	*P*
Time	14	294	6.97	***	6.64	***
Dispersal	2	42	92.77	***	834.85	***
Planting	1	21	10.42	**	25.62	***
Time*Dispersal	28	588	0.77		3.39	***
Time*Planting	14	294	0.48		0.36	
Dispersal*Planting	2	42	14.91	***	26.04	***
Disp*Time*Planting	28	588	0.26		0.46	
Recruit density	num.d.f.	den.d.f.	F-value	*P*	F-value	*P*
Time	14	294	8.05	***	9.31	***
Dispersal	2	42	118.25	***	820.48	***
Planting	1	21	28.57	***	21.71	***
Time*Dispersal	28	588	1.35		6.33	***
Time*Planting	14	294	1.19		1.04	
Dispersal*Planting	2	42	38.16	***	44.09	***
Disp*Time*Planting	28	588	0.14		0.89	

*P*<0.005 **,

*P*<0.0001 ***.

Fixed effects are dispersal mode (bird, bat or both) and planting treatment (planted or unplanted control), with time (16 to 76 months) after cattle exclusion.

Patterns of recruitment of later-successional species differed dramatically by dispersal category. Species densities of later-successionals increased four-fold from the initial to the final census (from 0.0005 m^−2^ in 2007 to 0.0023 m^−2^ in 2013, *P*<0.0001). At 76 months after cattle exclusion, species densities of later-successional trees and shrubs dispersed primarily by birds were five times higher than those dispersed by both bats and birds, with a significant interaction of dispersal category with time (Fig. 1*B, P*<0.0001). Later-successional species dispersed by bats alone were not present. Recruitment of species dispersed by birds appeared to inflect upward 60 months after cattle exclusion, and showed a strong and statistically significant inflection upward at 70 months as many planted animal-dispersed trees matured and bore fruit. The number of individual later-successional recruits increased ∼ seven-fold over the same period (0.0018 recruits m^−2^ to 0.012 m^−2^, *P*<0.0001), with a significant interaction of dispersal category with time (Fig. 1*D, P*<0.0001). Significant upward inflection of individual recruits dispersed by birds was evident at 48 months after cattle exclusion and increased dramatically with time as planted trees matured.

### Initial and final recruit densities

**Figure 2:**
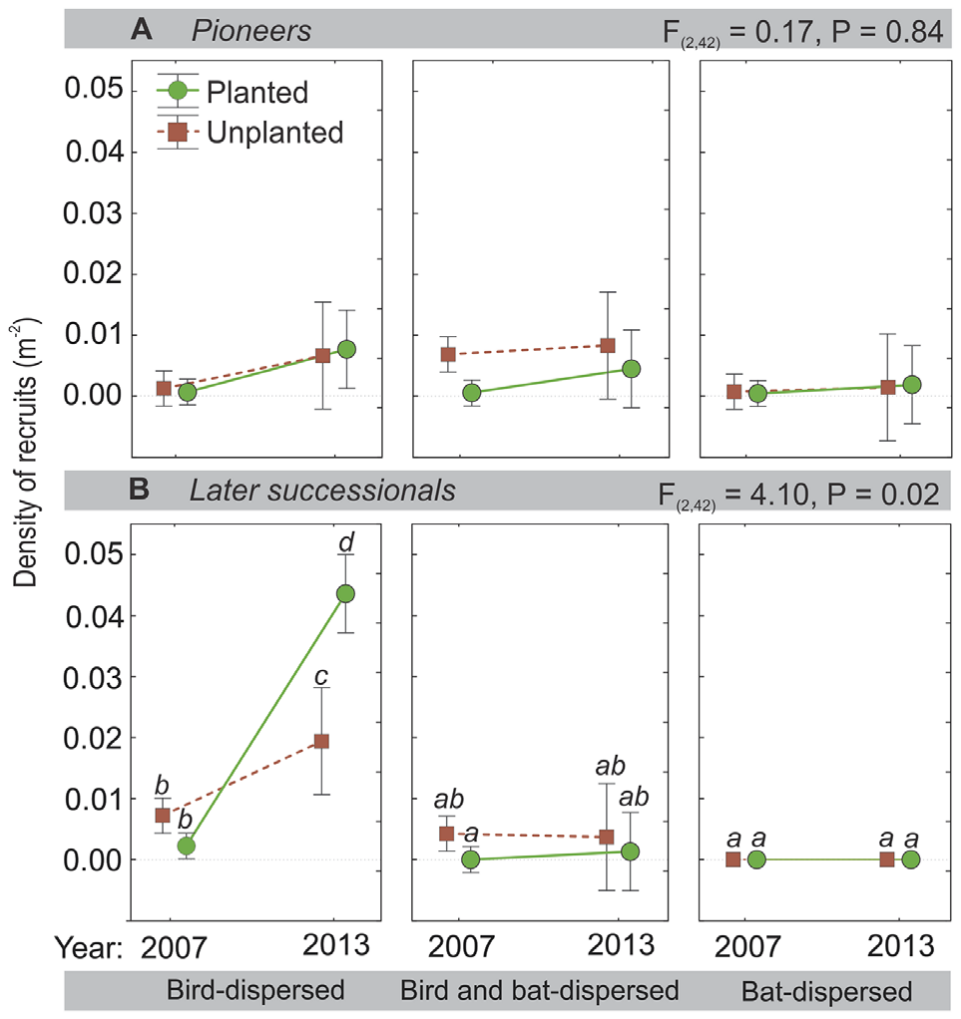
Net change in densities of recruited individuals by successional status and dispersal mode. Dispersal mode is indicated for (*A*) densities of pioneers and (*B*) densities of later-successional recruits. Blue circles indicate plantings, green squares unplanted controls. ANOVA statistics indicate disperser category by time interaction. Letters indicate differences between recruits in 2007 as compared with 2013 (Bonferroni adjustments, *P*<0.05). Shown are means and 95% confidence intervals.

The net contribution to recruitment of surviving individuals of pioneer and later-successional species dispersed primarily by birds was substantial, whereas the net effect of recruited species dispersed by both birds and bats or primarily by bats alone was not (Fig. 2, Table S1). Individual recruit densities of pioneer tree and shrub species dispersed by both birds and bats or bats alone differed little between 16 and 76 months after cattle exclusion, while pioneers dispersed by birds increased in both planted and control plots (Fig. 2*A*). Recruits of later-successional species dispersed by birds alone increased in both planted and control treatments, with the most dramatic increases in planted plots (Fig. 2*B*).

## Discussion

Tropical forests generate and maintain most of the biodiversity of terrestrial ecosystems on Earth [29], yet are rapidly succumbing to habitat loss as forested land is converted to pasture and crops [30], [31]. Less appreciated is re-forestation of land that is at least temporarily useless for agriculture [32], [33]. For land under human domination, reasonable priorities for conservation and restoration of tropical biodiversity include restoring dispersal processes that: (a) conserve biological diversity in highly altered agricultural landscapes by maintaining habitat heterogeneity, and (b) accelerate recovery of biodiversity and ecosystem services when land is abandoned to secondary succession.

Planned heterogeneity in agricultural landscapes contributes to both connectivity and recovery after abandonment. When land is too valuable to be released from agriculture, tree islands maintain substantial biological diversity and connectivity between large forest remnants, fragments, and restorations undergoing succession [23], [34]–[36]. Ultimately, mixed stands of reproductive trees serve as regeneration nuclei for forest recovery if depleted agricultural land is abandoned [37], [38]. Here we report roles of birds and bats that mediate colonization of experimental controls and plantings by tree species other than those we planted.

Later-successional trees rarely establish from seed in early successional habitats unless agents of dispersal are attracted to sites that coincidently have shade, moisture and protection from livestock [9]. Fenced and planted plots offer conditions that increase chances of germination and growth [15], [39]. It is also likely that scattered incoming recruits establish under a broader range of conditions than they would in closed forests. In early successional habitats, shade-tolerant species arrive by chance and establish and grow where it is physically possible, without much influence of competition from conspecifics until self-thinning occurs at sapling or young-adult stages. Until then many recruited species experience the advantage of few nearby conspecific competitors [40]. In a managed setting intended to optimize diversity of pollen and seed movement among plots and fragments over 20–30 years, trees will grow large enough to reproduce, but are unlikely to reach a size sufficient to prevent several other species from reaching maturity in a given plot.

In our experiment, influx of species from the landscape was notable. Ninety-four percent of animal-dispersed recruits that survived to 76 months were of species other than those that we planted, including 17 later-successional and 12 of 14 pioneer species. Most pioneer recruits in experimental plots were of shrubs and trees that produced fruits eaten by a wide variety of birds, bats, and terrestrial mammals. Pioneer recruits of *Cecropia obtusifolia, Conostegia xalapensis*, and *Witheringia nelsonii* evidently established from seeds brought by opportunistic bird or bat foragers on fruits of naturally regenerating *C. xalapensis* and planted *C. obtusifolia* and *Ficus yoponensis*. The latter two are heavily used by both birds and bats [8], [16], [41], [42]. More species of fruit-eating birds than fruit-eating bats occur in the Los Tuxtlas landscape, but it is still remarkable that seedling recruits of later-successional shrubs and trees were of species dispersed primarily by birds, and to a lesser extent by both birds and bats. None were dispersed by bats alone. Densities of some pioneer recruits dispersed primarily by birds increased slightly over time in both planted and control plots; densities of later-successional trees dispersed primarily by birds increased significantly in controls and dramatically in planted plots.

Use of fruiting trees by bats and birds is to a degree context-specific. *Ficus yoponensis*, a planted free-standing fig in our experiment with fruits available on at least three small adult trees within 48 months after cattle exclusion, is a “bat fruit” in diverse Central American forests [42]. The species is heavily used by both birds and bats in disturbed settings: as many as 45 species of fruit-eating birds forage in isolated *F. yoponensis* at Los Tuxtlas [16]. From 48–76 months after cattle exclusion, 5–36 individual planted *Cecropia obtusifolia* produce fruit at any given time. Some congeners of this species are more bat- than bird-dispersed (e.g. *C. peltata* versus *C. obtusifolia* in reference [41], *χ* ^2^ = 56.0, df = 1, *P*<0.001). In pastures at Los Tuxtlas, *C. obtusifolia* is both bird- and bat-dispersed [7], while in forest a menagerie of arboreal mammals also eat the fruits [43].

In the present experiment, legacy effects faded over time. A few older recruits, including reproductive *C. obtusifolia* and *Trema micrantha* and sapling *Bursura simaruba*, appeared in the plots directly after fencing, reflecting legacies of seeds brought to figs or other fruiting trees that were cut during site preparation [28]. Additional recruits appeared several months to years after fencing, well beyond dormancy periods for most Neotropical tree seeds [44], [45]. With either immediate or delayed germination, seedling survival poorly reflected seed arrival; germination, establishment, and recruitment to seedling and later life-history stages were context-dependent [4], [11], [14], [46]–[48]. For seeds that did arrive, xeric conditions and thick grass suppressed establishment of tree seedlings, killed seedlings that did establish, and likely intensified density-dependent mortality of seeds and young seedlings under perches.

Planted stands reduce both seed and establishment limitation. Birds that potentially disperse seeds are more likely to forage in clumps of shrubs or trees than in open areas, stay longer in larger than smaller stands of woody vegetation, and regurgitate or defecate more seeds in tree islands than in the open [34]–[36], [48], [49]. Moreover, seedlings fare better in shade where grass is at least partially suppressed than in the open [15], [40]. As expected, greater seed arrival and enhanced seedling survival substantially accelerate succession in planted stands as compared with unassisted natural succession in controls. Over the first six to seven years of restoration in the Los Tuxtlas landscape, fruit-eating birds are far more effective mediators of succession by later-successional trees and shrubs from forest than fruit-eating bats.

Only a small proportion of seeds resulted in established seedlings in pasture plots. Early in the present experiment, we reported seed input of woody plants into fenced plots >90 m from forest of one seed in 10 m^−2^ month^−1^ [12], a rate that increased substantially as succession occurred (de la Peña-Domene, unpublished data). After 76 months of cattle exclusion, actual recruit density in controls averaged ∼ one shrub or tree recruit 100 m^−2^, indicating immense mortality of seeds or young seedlings. In contrast, planted plots averaged ∼ one recruit 16 m^−2^. These densities were well below those under large fenced figs in a pasture landscape [49], and were below what would be expected in a mature closed-canopy continuous forest [50]. After more than six years of succession in cattle exclosures, recruited seedlings were still sparse.

With the exception of common pioneers, species planted in our experiment are in higher densities than occur in nature. High densities differentially affect survival of planted species, and will likely bias early recruitment in favor of species *other* than those that we planted [51], [52]. In the 50 ha Barro Colorado Island forest-dynamics plot, for instance, interactions among trees range from little thinning of older juveniles and adults to strong negative density-dependence among conspecifics, but with much less sensitivity to proximity of heterospecifics [40], [53]. In our experiment, we expect substantial thinning of saplings and young adults of most planted species. High densities of most planted species are also likely to directly or indirectly impede recruitment of conspecific seedlings.

Scattered colonists from forests will likely have a different dynamic. Plots with 120 m of edge admit diffuse light, explaining high establishment of pioneers during the first five years of cattle exclusion [15]. A question is whether deepening shade will repress recruitment of pioneers and allow later-successional species to establish in small (900 m^2^) stands. At least to established seedling, sapling and in some case adult stages, this is occurring, with a clear increase in establishment of later-successional species between 60 and 76 months, and little recruitment of most planted or early-recruited pioneers that are fruiting in planted plots.

It is too early to know whether the imbalance of bird- versus bat-dispersed later-successional recruits will persist. In addition to dispersal of small-seeded pioneers, bats disperse many large-seeded, late-successional species in continuous forest in both the Old- and New-World Tropics [54]–[56]. Large neotropical fruit bats (e.g. *Artibeus lituratus*, up to 70 g) carry *Dipteryx* (Fabaceae) fruits weighing >20 g for hundreds of meters, but even small fruit bats (e.g. *Artibeus watsoni*, 12 g) disperse a variety of tree seeds >20 mm long in extensive forest [57]. Fruit-eating bats large enough to carry fruits of substantial size (1–20 g) are common in the Los Tuxtlas landscape [7], [25]. Bats may play a greater role in recruitment of later-successional trees as planted figs mature and continue to grow in size and fecundity. The absence of a substantial role of dispersal of later-successional trees by bats during the first six years of experimental restoration is surprising.

Our study has significant implications for maintaining biodiversity in human-dominated landscapes. Mixed-species plantings potentially maintain and locally increase “countryside diversity” of tropical plants and animals that are capable of persisting in or moving through human-dominated landscapes [10], [23], [25], [58], [59]. Managed landscape heterogeneity is not a panacea for preservation of all tropical diversity; some fruit-eating animals fail to reach or thrive in forest islands, with adverse consequences for those tree species that depend on them [60]. Extensive remnants preserve tropical flora and fauna that require old-growth habitats, and preserve sources of plant and animal colonists of land that is eventually released from crops and pasture [61]. For the half of the rainforest biome that has been deforested during the last century, however, the goal of preserving all rainforest biodiversity is unrealistic. Many rare species with limited geographical distributions are almost certainly already extinct [62]. But others of conservation interest are potentially spared by heterogeneous habitats. For instance, in our experiment 38 well-established (25–52 cm tall) seedlings of endemic IUCN red-listed vulnerable *Ocotea uxpanapana* (Lauraceae) are present, as are 20 well-established (14–800 cm tall) *Tetrorchidium rotundatum* (Euphorbiaceae; de la Peña-Domene, unpublished data), a widespread animal-dispersed tree that is nonetheless endangered in Mexico. A realistic goal for maintenance and recovery of substantial tropical biodiversity is to sustain and re-establish dispersal processes that preserve or create as much habitat heterogeneity and connectivity as rural economies permit.

Habitat islands left as remnants or planted as stepping stones in matrices between forest remnants serve a variety of functions. Connectivity preserves landscape species richness at large scales [63]–[65]; stepping-stone forest islands retain and restore some tree species, serve as foraging and breeding refuges of mobile animals, contribute reproductive connectivity among trees through pollen exchange and seed dispersal, and provide nuclei of forest regeneration if land is released from agriculture. To date, increased understanding of the importance of seed dispersal by animals has had little effect on conservation or restoration in the tropics [59]. Creating heterogeneity with corridors and stepping-stone tree islands cannot preserve all tropical diversity; a key objective should be to retain as many uniquely important tree species and dispersal agents as possible. With half of the tropical rainforest biome cleared at least once in the last 100 years, forest conservation and restoration using birds and mammals that transport seeds should become a central theme in ecology of this century.

## Supporting Information

Table S1 **Dispersal mode and successional status of recruited species, including references for published reports.**
(DOC)

Appendix S1 **Criteria for designating dispersal modes and successional status of plants.**
(DOC)

Data Spreadsheed S1 **Data used for analyses.**
(XLSX)
