# Supplementary material for: Roles of Birds and Bats in Early Tropical-Forest Restoration
Source: PLoS One. 2014 Aug 13;9(8):e104656. doi: 10.1371/journal.pone.0104656 (PMC4131887; doi:10.1371/journal.pone.0104656)
Supplement: Table S1 — Dispersal mode and successional status of recruited species, including references for published reports. (DOC) [file pone.0104656.s001.doc]

**Table S1**. **Dispersal mode and successional status of recruited species, including references for published reports.** Designations of dispersal agents are generally consistent with recognized dispersal syndromes [1-3], indicated in italics. Field confirmations with direct observations of plants identified to the genus level are numbered in regular font; field confirmations identified to species are in bold. Ambiguous identifications of plants are checked with the Mexican National Herbarium at Universidad Nacional Autónoma de México.

| Species (family) | Primary dispersal | Life form | References |
| --- | --- | --- | --- |
| **Later Successional** | | | |
| *Alchornea latifolia* Sw. (Euphorbiaceae) | Birds | Tree | *1,2,3*,**7,8**,9,10,11,12,13 |
| *Bursera simaruba* (L.) Sarg. (Burseraceae) | Birds | Tree | *1,2,3*,**4,7,**11,**14** |
| *Clusia flava* Jacq*.* (Clusiaceae) | Birds | Tree | *1,3*,7,8,11,13,15,16 |
| ^a^ *Coccoloba hondurensis* Lundell (Polygonaceae) | Both | Tree | *2,3*,6,11,16,17,18 |
| *Cupania glabra* Sw*.* (Sapindaceae) | Birds | Tree | *1,2,3*,**4**,5,7,9,11,13,**16** |
| *Cymbopetalum bailonii* R. E. Fr. (Annonaceae) | Birds | Tree | *1,2,3*,**4**,14,**19** |
| ^b^ *Ficus* *aurea* Nutt. (Urostigma, Moraceae) | Both | Tree | *2,3*,**5,**7,9,10,11,12,13,15,  16,17,18,**20,**21,22,see 4 |
| ^c^ *Nectandra ambigens* (S.F. Blake) C.K. Allen (Lauraceae) | Birds | Tree | *2,3*,9,10,11,16 |
| *Ocotea uxpanapana* T. Wendt & van  der Werff (Lauraceae) | Birds | Tree | *2,3*,7,11,13,16 |
| ^d^ *Pleuranthodendron lindenii* (Turcz.) Sleumer (Salicaceae) | Birds | Tree | *1,2,3*,11,12,16 |
| ^e^ *Pseudolmedia oxyphyllaria* Donn. Sm.  (Moraceae) | Both | Tree | *2,3***,22**,23 |
| *Psychotria limonensis* K. Krause (Rubiaceae) | Birds | Shrub | *1,2,3*,9,11,12,13,15,  16,**24**,25,26 |
| *^f^ Rollinia jimenezii* Saff. (Annonaceae) | Both | Tree | *1,2,3*,11,**18** |
| ^g^ *Sapindus saponaria* L. (Sapindaceae) | Both | Tree | *2*,6,11,**17** |
| ^h^ *Sapium nitidum* (Monach.) Lundell (Euphorbiaceae) | Birds | Tree | *1,2,3*,11,16,28,29 |
| *Tabernaemontana* *alba* Mill. (Apocynaceae) | Birds | Tree | *1,2,3*,11,16 |
| *Tetrorchidium rotundatum* Standl.(Euphorbiaceae) | Both | Tree | *2,3*,**4**,15 |
| *Trichilia martiana* C. DC. (Meliaceae) | Birds | Tree | *1,2,3*,11,16,30,31,32 |
| *Trophis mexicana* (Liebm.) Bureau (Moraceae) | Birds | Tree | *1,2,3*,11,14,**16,**23 |
|  |  |  |  |
| **Pioneer** | | | |
| *Acacia cornigera* (L.) Wild. (Fabaceae) | Birds | Both | *2,3*,33 |
| *Cecropia obtusifolia* Bertol. (Cecropiaceae) | Both | Tree | *2,3*,**4,5,**6,7,8,9,**10**,12,13,**16**,  17**,18**,**21,22,34,35-38** |
| *Cestrum racemosum* R. & P.(Solanceae) | Birds | Tree | *1,2,3*,9,**10**,11,**16**,  cf.18,38 |
| *Clidemia* spp. (Melastomataceae) | Birds | Shrub | *1*,6,10,11,15,25,26 |
| *Conostegia xalapensis* (Kunth H.B.K.) G. Don ex DC. (Melastomataceae) | Both | Both | *2,3***,4**,10,11,**15,16,**  26**,34**,36,**39** |
| *Hampea nutricia* Fryxell (Malvaceae) | Birds | Tree | *1,2,3*,10,11,16,36 |
| *Miconia* spp. (Melastomaceae) | Birds | Both | *1*,7,8,9,10-13,15,  16,24-26,32,36,40 |
| *Piper amalago* L. (Piperaceae) | Bats | Tree | *1,2,3*,4,5,**6**,11,16,17,21,  **22**,35,36,**37**,38,**41,42** |
| *Piper hispidum* Sw. (Piperaceae) | Bats | Both | *1,2,3*,4,**5**,6,11,16,17,21,  **22**,35,36,37,**38** |
| ^i^ *Piper umbellatum* L. (Piperaceae) | Bats | Shrub | *2*,**4,5**,6,11,**16**,17,21,**22,**  **26,35**,36**,37,38** |
| ^j^ *Psidium guajava* L. (Myrtaceae) | Both | Tree | *1,3*,**6,**11,**17,35,44-47** |
| *Stemmadenia donnell-smithii* (Rose) Woodson (Apocynaceae) | Birds | Tree | *1,2,3***,**11,16**,48,49,50** |
| ^k^ *Trema micrantha* (L.) Blume (Ulmaceae) | Birds | Tree | *1,2*,**8**,11,**13,16**, **51,** cf. **4,22** |
| *Witheringia nelsonii* (Fern.) Hunz. (Solanaceae) | Both | Shrub | **5**,10,11,15,16,36,  **38**,52,53 |
| ^a^ An unusual compilation of difficult-to-find literature on bat frugivory is Lobova et al. (18).  ^b^ syn. *Ficus tecolutensis* (Liebm.) Miq.  ^c^ Sometimes eaten by large bats (*Artibeus jamaicensis*, 22).  ^d^ Included here is closely-related (*Hasseltia*,11,14,16) with broad avian disperser assemblages.  ^e^ Mostly primates in forest (e.g. 22), which are not present in pasture plots.  ^f^ syn. Rollinia mucosa (Jacquin) Baillon.  ^g^ Direct observations are scarce. Note 27.  ^h^ syn. for *Sapium sebiferum* (L.) Roxb. is *Triadica sibifera* (L.) Small.  ^i^ Included with syn. *Pothomorphe umbellata* (L.) Miqu. in a tight *Piper auritum* clade (43). *Piper auritum* fruits are eaten by birds (16,26,36), but where direct quantitative comparisons are available seeds are overwhelmingly dispersed by bats (4,5).  ^j^ This is an extreme generalist, with records including birds, bats, other mammals, and reptiles  (48).  ^k^ Bats sometimes eat this fruit (4,22), but it appears to be mostly bird-dispersed. | | | |
|  | | | |

**Table S1 references**

1. Van der Pijl L (1982) Principles of dispersal in higher plants. 3rd edition. Berlin: Springer-Verlag.

2. Ibarra-Manriquez G, Martinez-Ramos M, Oyama K (2001). Seedling functional types in a lowland rain forest in Mexico. American Journal of Botany 88(10):1801-1812.

3. Guevara S, Meave J, Casasola PM, Laborde J, Castillo S (1994) Vegetacion y flora de

potreos en la Sierra de los Tuxtlas, Mexico. Acta Botanica Mexicana 28: 1-27.

4. Galindo-Gonzalez J, Guevara S, Sosa VJ (2000) Bat- and bird-generated seed rains at isolated trees in pastures in a tropical rainforest. Conservation Biology 14: 1693–1703. 5. Medellin R A, Gaona O (1999) Seed dispersal by bats and birds in forest and disturbed habitats of Chiapas, Mexico. Biotropica 31(3): 478-485.

6. Fleming TH (1988) The short-tailed fruit bat. Chicago: University of Chicago Press.

7. Carlo TA, Collazo JA, Groom MJ (2003) Avian fruit preferences across a Puerto

Rican forested landscape: Pattern consistency and implications for seed removal.

Oecologia 134: 119-131.

8. Skutch AF (1980) Arils as food of tropical American birds. Condor 82(1): 31– 42.

9. Kessler-Rios MM, Kattan GH (2012) Fruits of Melastomataceae: phenology in Andean forest and role as a food resource for birds. Journal of Tropical Ecology 28: 11-21. 10. Blake JG, Loiselle BA (1992) Fruits in the diets of neotropical migrant birds in Costa Rica. Biotropica 24(2a):200-210.

11. Snow DW (1981) Tropical frugivorous birds and their food plants: A world survey.

Biotropica 13(1):1–4.

12. Greenberg R (1981) Frugivory in some migrant tropical forest wood warblers.

Biotropica 13(3): 215-223.

13. Galetti M, Laps R, Pizo MA (2000) Frugivory by toucans (Ramphastidae) at two

altitudes in the Atlantic forest of Brazil. Biotropica 32(4b): 842-850.

14. Foster MS (2007) The potential of fruit trees to enhance converted habitats for

migrating birds in southern Mexico. Bird Conservation International 17: 45-61.

15. Boyle WA, Conway CJ, Bronstein JL (2011) Why do some, but not all, tropical birds migrate? A comparative study of diet breadth and fruit preference. Evolutionary

Ecology 25: 219–236.

16. Wheelwright NT, Haber WA, Murray KG, Guindon G (1984) Tropical fruit-eating

birds and their food plants – a survey of a Costa Rican lower montane forest. Biotropica

16: 173-192.

17. Greenhall AM (1957) Food preferences of Trinidad fruit bats. Journal of Mammalogy

38(3): 409-410.

18. Lobova TA, Geiselman CK, Mori SA (2009) Seed Dispersal by Bats in the Neotropics

(New York Botanical Society, New York).

19. Coates-Estrada R, Estrada A (1988) Frugivory and seed dispersal in *Cymbopetalum*

*baillonii* (Annonaceae) at Los Tuxtlas, Mexico. Journal of Tropical Ecology 4: 157-162.

20. Guevara S, Laborde J (1993) Monitoring seed dispersal at isolated standing trees in

tropical pastures: consequences for local species availability. Vegetatio107/108: 319-338.

21. Bonaccorso FJ (1979) Foraging and reproductive ecology in a Panamanian bat

community. Bulletin of the Florida State Museum, Biological Sciences 24: 359-408.

22. Estrada A, Coates-Estrada R, Vasquez-Yanes C, Orozco-Segovia A (1984) Comparison

of frugivory by howling monkeys (*Alouatta palliata*) and bats (*Artibeus jamaicensis*)

in the tropical rain forest of Los Tuxtlas, Mexico. American Journal of Primatology 7:

3-13.

23. Lancaster DA (1964) Life history of the Boucard tinamou in British Honduras Part I: Distribution and general behavior. Condor 66(3): 165– 181.

24. Poulin B, Wright SJ, LeFebvre G, Calderon O (1999) Interspecific synchrony and

asynchrony in the fruiting phenologies of congeneric bird-dispersed plants in Panama.

Journal of Tropical Ecology 15: 213-227.

25. Morales-Betancourt JA, Castano-Villa CJ, Fonturbel FE (2012) Resource abundance

and frugivory in two manakin species (Aves: Pipridae) inhabiting a reforested area in

Colombia. Journal of Tropical Ecology 28: 511-514.

26. Loiselle BA (1990) Seeds in droppings of tropical fruit-eating birds: importance of

considering seed composition. Oecologia 82: 494-500.

27. Carlquist S (1966) The biota of long-distance dispersal. III. Loss of dispersibility in the

Hawaiian flora. Brittonia 18(4): 310-335.

28. Renne IJ, Barrow W C., Jr., Randall LA., Bridges, WB, Jr (2002) generalized avian dispersal syndrome contributes to Chinese tallow tree (*Sapium sebiferum*,

Euphorbiaceae) invasiveness. Diversity and Distributions 8: 285–295.

29. Aslan CE (2011) Implications of newly-formed seed-dispersal mutualisms between birds and introduced plants in northern California, USA. Biological Invasions 13:

2829–2845.

30. Leck CF (1969) Observations of birds exploiting a Central American fruit tree.

Wilson Bulletin 81: 264-269.

31. Foster MS, McDiarmid RW (1983) Nutritional value of the aril of *Trichilia cuneata*, a bird-dispersed fruit. Biotropica 15(1): 26-31.

32. Poulin B, LeFebvre G, McNeil R (1992) Tropical avian phenology in relation to

abundance and exploitation of food resources. Ecology 73(6): 2295-2309.

33. Janzen DH (1966) Coevolution of ants and acacias in Central America. Evolution 20(3): 249-275.

34. Cole RJ, Holl KD, Zahawi RA (2010) Seed rain under tree islands planted to restore

degraded lands in a tropical agricultural landscape. Ecol Appl 20(5): 1255-1269.

35. Garcıa-Estrada C, Damon A, Sanchez-Hernandez C, Soto-Pinto L, Ibarra-Nunez G

(2012) Diets of frugivorous bats in montane rain forest and coffee plantations in

southeastern Chiapas, Mexico. Biotropica 44(3): 394 – 401.

36. Palmeirim JM, Gorchov DL, Stoleson S (1989) Trophic structure of a neotropical

frugivore community: Is there competition between birds and bats? Oecologia 79: 403-

411.

37. Dinerstein E (1986) Reproductive ecology of fruit bats and the seasonality of fruit production in a Costa Rican cloud forest. Biotropica 18(4): 307-318.

38. Goncalves da Silva AG, Gaona O, Medillin RA (2008) Diet and trophic structure in a

community of fruit-eating bats in Lacandon Forest, Mexico. Journal of Mammalogy 89(1): 43–49.

39. Castro-Luna AA, Vinicio J, Sosa VJ (2009) Consumption of *Conostegia xalapensis*

fruits and seed dispersal of *Coussapoa oligocephala* by the nectarivorous bat

*Hylonycteris underwoodi* Thomas, 1903 (Chiroptera: Phyllostomidae). Studies on Neotropical Fauna and Environment 44: 137-139.

40. Loiselle BA, Blake JG (1999) Dispersal of melastome seeds by fruit-eating birds of

tropical forest understory. Ecology 80(1): 330-336.

41. Fleming TH, Heithaus ER, Sawyer WB (1977) An experimental analysis of the food location behavior of frugivorous bats. Ecology 58(3): 619-627.

42. Fleming TH (1981) Fecundity, fruiting pattern, and seed dispersal in *Piper amalago*

(Piperaceae), a bat-dispersed tropical shrub. Oecologia 51: 42-46.

43. Jaramillo MA, Manos PS (2001) Phylogeny and patterns of floral diversity in the genus

Piper (Piperaceae). American Journal of Botany 88(4): 706–716.

44. Jordaan LA, Johnson SD, Downs CT (2011) The role of avian frugivores in germination of seeds of fleshy-fruited invasive alien plants. Biological Invasions 13: 1917–1930.

45. Jordaan, LA, Johnson SD, Downs CT (2012) Wahlberg’s epauletted fruit bat

(*Epomophorus wahlbergi*) as a potential dispersal agent for fleshy-fruited invasive alien

plants: effects of handling behaviour on seed germination. Biological Invasions 14: 959–968.

46. Berens DG, Farwig N, Schaab G, Bohning-Gaese KB (2008) Exotic guavas are foci of

forest regeneration in Kenyan farmland. Biotropica 40(1): 104-112.

47. Heleno RH, Olesen MN, Vargas P, Traveset A (2013) Seed dispersal networks in the Galápagos and the consequences of alien plant invasions. Proceedings of the Royal Society B 280: 20122112. <http://dx.DOI.org/10.1098/rspb.2012.2112>.

48. McDiarmid RW, Ricklefs RE, Foster MS (1977) Dispersal of *Stemmadenia donnell-smithii* (Apocynaceae) by birds. Biotropica 9: 9–25.

49. Coates-Estrada R, Estrada A,.Merritt D Jr (1993) Foraging by parrots (*Amazona autumnalis*) on fruits of *Stemmadenia donnell-smithii* (Apocynaceae) in the tropical rain forest of Los Tuxtlas, Mexico. Journal of Tropical Ecology 9: 121-124.

50. Shweiki S, Howe HF (2009) Regeneration of deep-forest *Stemmadenia donnell-smithii*

(Apocynaceae) at Los Tuxtlas, Mexico. Plant Species Biology 24: 225-228.

51. Vazquez-Yanes C (1998) *Trema micrantha* (L.) Blume (Ulmaceae): A promising neotropical tree for site amelioration of deforested land. Agroforestry Systems 40: 97-104.

52. Murray KG (1988) Avian seed dispersal of three neotropical gap-dependent plants. Ecological Monographs 58(4): 271-298.

53. Murray KG, Russell S, Picone CM, Winnett-Murray K, Sherwood W, Kuhlmann ML (1994) Fruit laxatives and seed passage rates in frugivores: Consequences for plant reproductive success. Ecology 75(4): 989-994.
