# Supplementary material for: Roles of Birds and Bats in Early Tropical-Forest Restoration
Source: PLoS One. 2014 Aug 13;9(8):e104656. doi: 10.1371/journal.pone.0104656 (PMC4131887; doi:10.1371/journal.pone.0104656)
Supplement: Appendix S1 — Criteria for designating dispersal modes and successional status of plants. (DOC) [file pone.0104656.s002.doc]

Appendix S1: **Criteria for designating dispersal modes and successional status.** Dispersal modes of species that recruit to our experimental plots are generally consistent with recognized suites of fruit characters: scentless “bird fruits” are colorful red, blue, yellow, black, or multicolored, while musty or otherwise odorous “bat fruits” are white, yellowish, or green [1]. These syndromes are also generally consistent with designations by botanists working in the Los Tuxtlas region [2, 3]. Because dispersal syndromes do not always predict actual use by fruit-eating animals in a given landscape, examples of direct observations are given to at least the genus level when observed usage confirms or contradicts expectations from syndromes. Records of direct use of species reflect different levels of accuracy. Most useful are unusual simultaneous comparisons of dispersal of plant taxa by birds and bats in southern Mexico [4, 5]. For instance, colorful fruits of strangler figs (subgenus Urostigma of *Ficus*) fit the bird-dispersed syndrome, while green fruits of free-standing figs (subgenus Pharmacoscya of *Ficus*) suggest the bat or arboreal mammal syndrome. Empirical collections from seed traps that distinguish diurnal from nocturnal seed deposition in southern Mexico indicate broad overlap of use of both fig subgenera by birds and bats [4, 5]. Moreover, many tree taxa recruited in our plots would additionally be dispersed by primates or by other arboreal or terrestrial mammals in continuous forest, but these potential dispersal agents are absent from plots (primates) or are unlikely to be frequent visitors that cross open pastures for the first years of this experiment before planted trees produce substantial quantities of fruit. We expect regular use by terrestrial omnivores that eat fruit and defecate intact seeds (*Canis latrans, Didelphis spp., Nasua narica, Procyon lotor*) when planted and eventually recruited animal-dispersed trees produce large enough fruit crops to draw the animals across open ground. Here, aside from the general treatise on dispersal syndromes [1] or supplements to botanical references [2, 3], we minimize compounding “syndrome errors” by citing representative studies that report data from direct observation of foraging animals, fecal samples, or from seed traps that distinguish day and night catches. Records that report animal use of the same species that we report are in bold type, those to the same genus in regular type. Many papers that cite use of a species indicate use of congeneric plants as well. Where quantitative comparisons are available with day and night measures of bird and bat use of tree and shrub species [4, 5], trees are considered “bird-” or “bat-” dispersed if the number of seeds in one category is > 4X seeds in the other category. In other cases we take the weight of evidence. For instance *Clidemia* is generally listed as bird-dispersed (Table S1). Occasional use of *C. octona* by bats is known (Appendix 8 of [7]). Designations of successional stage are open to interpretation. Early pioneer species colonize open areas and live up to 30 years (“tempranos”) or as late-pioneers up to 60 years (“tardios”) of Martinez-Ramos [6]. Most of these are listed as “pioneers” by Ibarra-Manriquez et al. [2], the term that we adopt. We use “later-successional” to conform to designations of “persistent” by Ibarra-Manriquez et al. [2] and “nomadas” and “tolerantes” of Martinez-Ramos [6]. Published designations of successional stage of *Piper amalago* and *Stemmadenia donnell-smithii* are contradictory; we regard them as pioneers because both are rapidly-maturing early colonists of open ground in our landscape. Both also occur in mature forest. In addition to the animal-dispersed species listed (Table S1), seedlings of 13 wind-dispersed trees and shrubs also recruited in plots. These included five planted species (*Albizia purpusii* Britton & Rose*, Cedrela ordorata* L.*, Heliocarpus appendiculatus* Turcz*, Ochroma pyramidale* (Cav. ex Lam.) Urb.*, Vochysia guatemalensis* Donn. Sm.), of which all but *C. odorata* seeded between 60 and 76 months after cattle exclosure. Other wind-dispersed recruits included five pioneers (*Eupatorium galeottii* B.L. Rob.*, Cnidoscolus multilobus* Pax. I. M. Johnston*, Heliocarpus donnell-smithii* Rose, *Lippia microphylla* Cham., *Trichospermum galeottii* (Turcz.) Kosterm) and two later-successional trees (*Cordia alliodora* (Ruiz & Pav.) Oken, *Dalbergia glomerata* Hemsl.). *Cordia alliodora* conspecifics were close to isolated pasture conspecifics and stumps of conspecifics cut during site preparation.

**Appendix S1 references**

1. Van der Pijl L (1982) Principles of dispersal in higher plants. 3rd edition. Berlin: Springer-Verlag.

2. Ibarra-Manriquez G, Martinez-Ramos M, Oyama K (2001). Seedling functional types in a lowland rain forest in Mexico. American Journal of Botany 88: 1801-1812.

3. Guevara S, Meave J, Casasola PM, Laborde J, Castillo S (1994) Vegetacion y flora de

potreos en la Sierra de los Tuxtlas, Mexico. Acta Botanica Mexicana 28: 1-27.

4. Galindo-Gonzalez J, Guevara S, Sosa V J (2000) Bat- and bird-generated seed rains at

isolated trees in pastures in a tropical rainforest. Conservation Biology 14: 1693–1703.

5. Medellin R A, Gaona O (1999) Seed dispersal by bats and birds in forest and disturbed

habitats of Chiapas, Mexico. Biotropica 31: 478-485.

6. Martínez-Ramos M (1985) Claros, ciclos vitales de los arboles tropicales y regeneración natural de las selvas altas perennifolias. In: Gómez-Pompa A, Del-Amo S, editors. Investigaciones sobre la regeneración de selvas altas en Veracruz, México. Mexico City: Alhambra Mexicana S.A. de C.V. pp 191-240.

7. Fleming TH (1988) The short-tailed fruit bat. Chicago: University of Chicago Press.
